# From Tokenization to Self-Supervision: Building a High-Performance Information Extraction System for Chemical Reactions in Patents

**DOI:** 10.3389/frma.2021.691105

**Published:** 2021-12-22

**Authors:** Jingqi Wang, Yuankai Ren, Zhi Zhang, Hua Xu, Yaoyun Zhang

**Affiliations:** ^1^ Melax Technologies, Inc., Houston, TX, United States; ^2^ School of Medicine, Nantong University, Nantong, China; ^3^ School of Biomedical Informatics, University of Texas Health Science Center at Houston, Houston, TX, United States

**Keywords:** named entity recognition, relation extraction, chemical reaction, chemical patent, tokenization for chemical patent, self-supervision, event extraction

## Abstract

Chemical reactions and experimental conditions are fundamental information for chemical research and pharmaceutical applications. However, the latest information of chemical reactions is usually embedded in the free text of patents. The rapidly accumulating chemical patents urge automatic tools based on natural language processing (NLP) techniques for efficient and accurate information extraction. This work describes the participation of the Melax Tech team in the CLEF 2020—ChEMU Task of Chemical Reaction Extraction from Patent. The task consisted of two subtasks: (1) named entity recognition to identify compounds and different semantic roles in the chemical reaction and (2) event extraction to identify event triggers of chemical reaction and their relations with the semantic roles recognized in subtask 1. To build an end-to-end system with high performance, multiple strategies tailored to chemical patents were applied and evaluated, ranging from optimizing the tokenization, pre-training patent language models based on self-supervision, to domain knowledge-based rules. Our hybrid approaches combining different strategies achieved state-of-the-art results in both subtasks, with the top-ranked F1 of 0.957 for entity recognition and the top-ranked F1 of 0.9536 for event extraction, indicating that the proposed approaches are promising.

## Introduction

New compound discovery plays a vital role in the chemical and pharmaceutical industry (Akhondi et al., 2019). Characteristics of compounds, such as their reactions and experimental conditions, are fundamental information for chemical research and applications (Akhondi et al., 2014). The latest information of chemical reactions is usually present in patents and is embedded in free text (Senger et al., 2015). The rapidly accumulating chemical patents urge automatic tools based on natural language processing (NLP) techniques for efficient and accurate information extraction (Muresan et al., 2011).

However, current NLP works in the biomedical domain mainly focus on chemical information extraction from biomedical literature, clinical text, or drug labels (Swain and Cole, 2016; Lowe, 2012; Wei et al., 2015; Xu et al., 2017). Plenty of shared tasks were organized, providing benchmark datasets, attracting active participations, and promoting state-of-the-art method and system development with community efforts (Wei et al., 2015; Xu et al., 2017). For example, the BioCreative V challenge was to identify chemical-induced disorders from biomedical literature (Wei et al., 2015); the task in N2C2 2018 extracts medicines, their attributes, and their adverse events from clinical text (Wei et al., 2019); TAC 2017 (Xu et al., 2017) and FDA 2019 (Bayer et al., 2020) also organized similar challenges to extract medical information from drug labels. As a unique genre different from other resources, patents have more timeliness, as an important knowledge source for new discoveries of chemical compounds (He et al., 2020). Despite increasing attention being paid to information extraction from chemical patents in recent years (Zhai et al., 2019; Hemati and Mehler, 2019), only one shared task in BioCreative V was organized to extract chemical and protein information from patent abstracts (Krallinger et al., 2015a). Lack of benchmark data with annotations of detailed information critical for new chemical compound discovery hinders the development of NLP-based information extraction systems that can be applied in a real-world setting.

Fortunately, the CLEF 2020—ChEMU Task takes the initiative to promote the chemical reaction extraction from patents by providing benchmark annotation datasets. Two important subtasks are set up in this challenge: chemical named entity recognition (NER) and chemical reaction event extraction. In particular, the annotation scheme of this benchmark data extends from previous challenges of chemical information extraction^1514^ to recognize multiple semantic roles of chemical substances in the reaction. Moreover, keyword event triggers and their relations with each semantic role are also annotated and provided for this task. The CLEF 2020—ChEMU Task will greatly facilitate the development of automatic NLP tools for chemical reaction in patents with community efforts (Nguyen et al., 2020; He et al., 2020).

At present, it is well acknowledged that deep learning-based algorithms have achieved state-of-the-art performances on various tasks of biomedical information extraction, which especially benefit from language models pre-trained on large-scale unlabeled text (Lee et al., 2020). Representative pre-trained language models include BERT (Devlin et al., 2018), RoBERTa (Liu et al., 2019), Electra BERT (Clark et al., 2020), and others built from open domain text and BioBERT (Lee et al., 2020), Clinic BERT (Alsentzer et al., 2019), and others built from fine-tuning BERT on large-scale biomedical text. These works lay a solid foundation of applying NLP to chemical patents. However, there are still some challenges in building high-performance systems for extracting information from chemical patents.

### Poor Output From Tokenization

Most previous works directly applied the original tokenizer WordPiece in BERT to preprocess the text input (Summary of the tokenizers, 2021), which was built on open text and not sufficient to interpret and represent mentions of biomedical concepts such as chemicals and numeric values. Mentions of such concepts have unique patterns and are common as semantic roles in chemical reactions, such as chemicals, temperatures, and percentages. However, few efforts have been made to optimize the output from tokenization, which plays an essential role in accurate extraction of elements in chemical reactions.

### Lack of Patent Language Models

The advantages of a pre-trained language model rely on the distributional semantic representation automatically learned to feature different genres of text. However, the existing biomedical language models mainly use biomedical literature or clinical text for pre-training,^1721^ rather than patents, for chemical information extraction.

### Uncovered Domain Knowledge

The current frameworks of language models are built on sentences, which are not sufficient to cover the long-distance patterns in documents, especially the domain knowledge related to the logical organization of patent structure (USPTO, 2021).

To address the above challenges and build a high-performance end-to-end system for extracting chemical reactions from patents, we participated in both subtasks in CLEF 2020—ChEMU and developed hybrid approaches that combined multiple strategies for performance enhancement. Our approaches achieved top rank in both subtasks, indicating that the proposed approaches are promising (He et al., 2020; He et al., 2021; Zhang and Zhang, 2020). The major contributions of this work are threefold, as summarized below:

1. We examined the output from tokenization and its effects on information extraction carefully and then tuned the tokenizer to get a more appropriate input for training deep learning models, which improved the performance significantly.
2. Pre-trained language models were built for chemical patents in a self-supervision way by fine-tuning BioBERT using the training and development data of ChEMU and chemical patents collected externally. Word2vec embeddings of chemical patents provided in the ChEMU Task (He et al., 2020), and the two pre-trained language models were compared for performance improvement.
3. Domain knowledge-based patterns were further summarized, and pattern-based rules were added into the NLP pipeline to further boost the performance.

## Materials and Methods

### Materials

#### Annotations of Chemical Reaction

**FIGURE 1:**
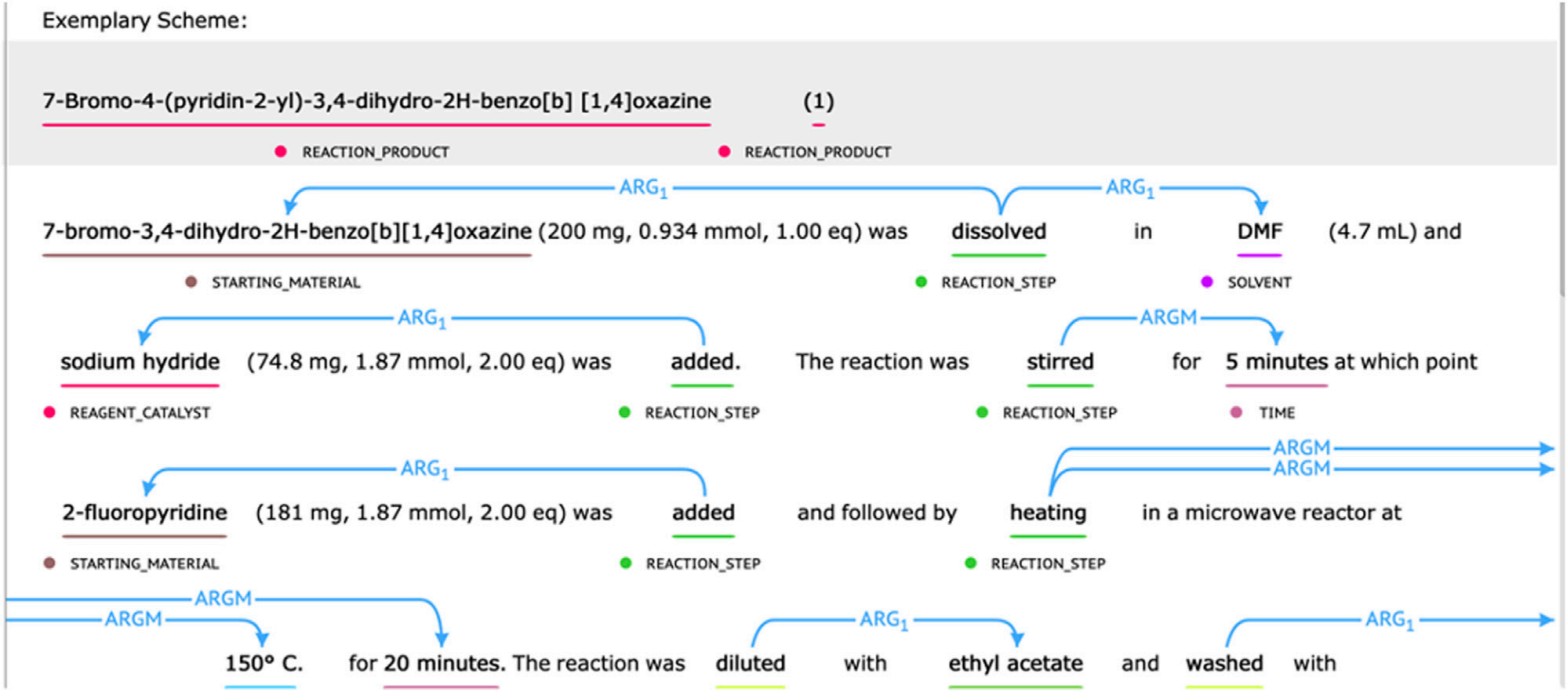
Examples of chemical reaction elements and relations annotated in patents.

The chemical reaction corpus provided by the CLEF 2020—ChEMU Task contained 1,500 selected patent snippets (He et al., 2020). It was split into training data, development data, and test data with a ratio of 0.6/0.15/0.25 (He et al., 2020). For subtask 1, it was annotated with 10 entity-type labels describing different semantic roles in chemical reaction, including EXAMPLE_LABEL, STARTING_MATERIAL, REAGENT_CATALYST, REACTION_PRODUCT, SOLVENT, TIME, TEMPERATURE, YIELD_PERCENT, YIELD_OTHER, and OTHER_COMPOUND. For subtask 2, the event trigger words (such as “addition” and “stirring”) were annotated, which were further split into labels of “REACTION_SETUP” and “WORK_UP.” Their relations with different semantic roles were also annotated. Following the semantic proposition definition, the Arg1 type was used to mark the relation between event trigger words and compounds. ArgM represented the auxiliary role of the event and was used to mark the relation between the trigger word and the temperature, time, or output entity (He et al., 2020). Some annotation examples of chemical reactions are displayed in Figure 1.

#### External Dataset of Chemical Patents

A set of additional patent snippets with chemical reactions was also collected by searching the Google Patent portal. First, we used generic keywords to form the initial query “(chemical) AND (compound) AND [(reaction) OR (synthesis)].” The IPC subclasses (Akhondi et al., 2019) most frequently occurred with chemical compounds, and reactions were used to further limit the scope of patents—A61K, A61B, C07D, A61F, A61M, and C12N (https://www.wipo.int/classifications/ipc/en/). In addition, following the data source constraints in ChEMU 2020, the patent language was restricted to English, and the patent sources were restricted to the European Patent Office and the United States Patent and Trademark Office. Once the patents were retrieved, they were manually reviewed. Some common keywords such as “step,” “example,” “yield,” and “stirred” were used to quickly locate text describing chemical reaction processes. The text snippets were then manually reviewed to maintain correct boundaries. The text snippets in the training and development data were also compared with the newly collected ones to ensure no redundant text was saved. The total number of text snippets with chemical reactions in each relevant patent varied from 1 or 2 to more than 300. In the end, about 20,000 snippets of chemical reactions were collected. This dataset was used as an argument for the training and development data of ChEMU 2020, to generate language models of chemical patents.

### Information Extraction

**FIGURE 2:**
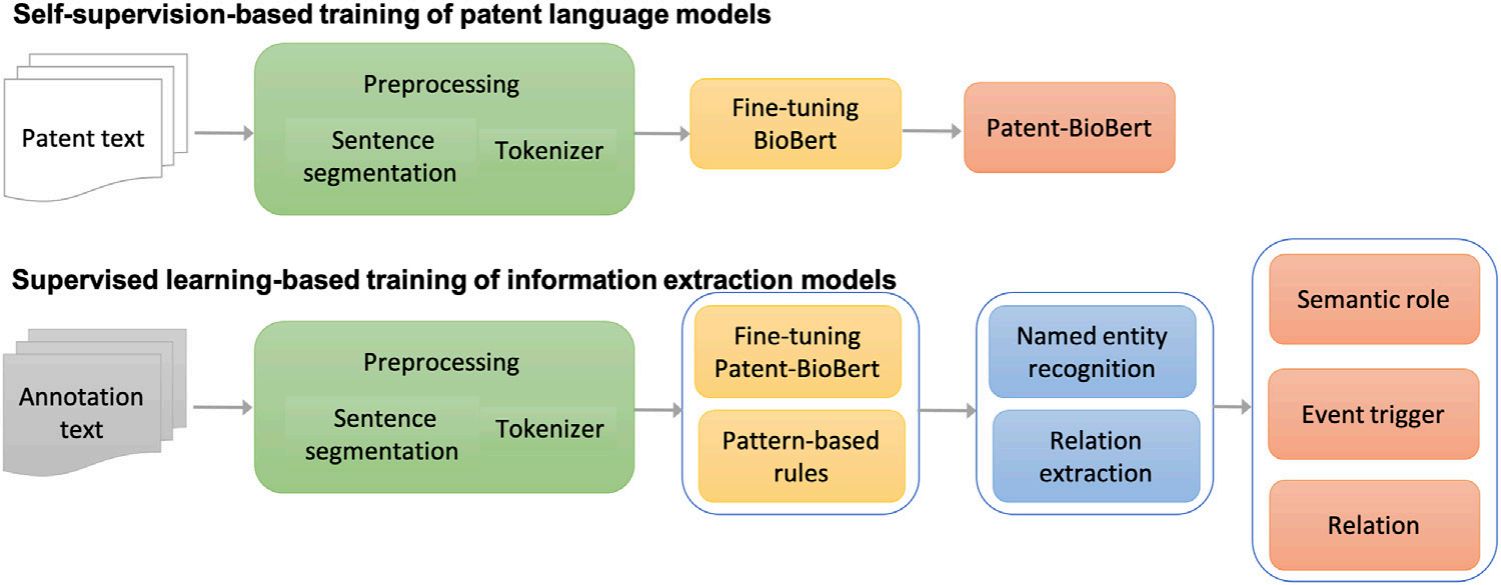
Workflow of building information extraction systems for chemical reactions in patents.

The major workflow of building information extraction systems for chemical reactions in patents is illustrated in Figure 2. The workflow consists of four steps:

(1) Text preprocessing: As a fundamental step in the pipeline, patent text was preprocessed to split sentences and tokens in each sentence. The sentence segmentation module and tokenizer in the CLAMP (Clinical Language Annotation, Modeling, and Processing Toolkit) toolkit were used in this step. The tokenizer was customized for chemical patent specifically.

(2) Pre-training of patent language models: Once processed, the patent text would be formatted as input to fine-tune the BioBERT language model generated from biomedical literature for patent text. Through this self-supervision, a patent language model was produced (named Patent_BioBERT).

(3) Model training for NER: After Patent_BioBERT was generated, it was further fine-tuned on the training and development data under the supervised learning framework to build models for NER. Mentions of semantic roles and event triggers of chemical reactions were considered as named entities and recognized by the trained models.

(4) Model training for relation extraction: Similarly, Patent_BioBERT was fine-tuned to build binary-classification models, which identified relations between semantic roles and event triggers.

(5) Rule-based postprocessing: Patterns not covered by the deep learning models, especially those within long distances, were summarized and tested carefully in a postprocessing step to further improve the performances.

Details of each step in the workflow are described as follows.

#### Pre-Processing

In this step, patent text was segmented into sentences through detecting sentence boundaries. Tokens in each sentence were also identified and separated by a tokenizer based on lexicons and regular expressions. Modules of sentence segmentation and tokenization in the CLAMP software (Soysal et al., 2018) were applied in this study. The sentence segmentation function in CLAMP was implemented using rules. It was designed specifically for biomedical text.

Notably, the tokenizer in CLAMP, a rule-based tool designed for biomedical text, was applied and revised to optimize the tokenization output. Our deep learning models using the default WordPiece tokenizer yielded modest NER performances. A careful observation showed that WordPiece could not split text with punctuations properly, especially for long-string mentions of chemical structures, as well as numerical expressions (e.g., “6.5 mg” and “2%”). To address this problem, we tried to simplify the tokenization process by taking every punctuation as a separator of tokens. For example, the sentence “7-Bromo-4-(pyridin-2-yl)-3,4-dihydro-2H-benzo(b) (1,4)oxazine (1)” will be tokenized into “7/-/Bromo/-/4/-/(/pyridine/-/2/-/yl/)/-/3/,/4/-/dihydro/-/2H/-/benzo/(/b/)//(/1/,/4/)/oxazine//(/1/)/” (the symbol “/” is used to separate tokens in this example). For this, we revised the tokenizer in CLAMP and applied it to each sentence first. After that, the WordPiece tokenizer would work on the input sentences and generate sub-words wherever necessary to be in line with the vocabulary. Therefore, a two-stage tokenization was implemented. Our assumption was that this process would produce tokens in a more consistent way, and it would be more convenient for the deep learning algorithms to use context information for boundary detection. As shown in the later sections of Results and Discussion, this strategy worked and improved the NER performance significantly.

#### Pre-training Language Model on Patents

Diverse expressions of chemical reaction information in the free text make them very sparse to be represented and modeled (Camacho-Collados and Pilehvar, 2018). The semantic distributed representations (i.e., multidimensional vectors of float values) of text generated by deep neural networks, or deep learning methods, alleviated the sparseness challenge by dramatically reducing the dimensions of language representation vectors using a nonlinear space (Camacho-Collados and Pilehvar, 2018). Specifically, language models pre-trained on large-scale unlabeled datasets embed linguistic and domain knowledge that can be transferred to downstream tasks, such as NER and relation extraction (Liu et al., 2016). BioBERT (Lee et al., 2020), a pre-trained biomedical language model (a bidirectional encoder representation for biomedical text), was used as the basis for training a language model of patents. Based on BERT (Devlin et al., 2018), a language model pre-trained on large-scale open text, BioBERT was further refined on using the biomedical literature in PubMed and PMC. Consequently, BioBERT outperforms BERT on a series of benchmark tasks for biomedical NER and relation extraction (Lee et al., 2020). For this study, BioBERT was retrained using text files provided by CLEF 2020—ChEMU and external text of chemical reactions manually collected from Google Patent Search to adapt the language model to patent data. For convenience, the pre-trained language model is named Patent_BioBERT.

#### Subtask 1—NER

**FIGURE 3:**
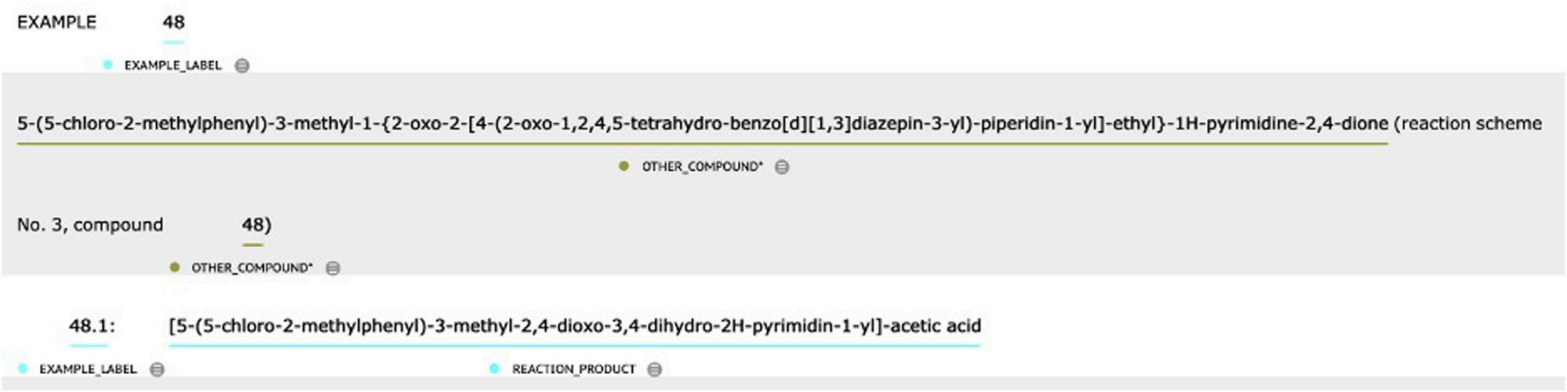
An example of a text snippet with hierarchical steps of chemical reactions.

Semantic roles in chemical reactions are recognized using a hybrid method. First, Patent_BioBERT was fine-tuned using the Bi-LSTM-CRF (Bi-directional Long–Short-Term-Memory Conditional-Random-Field) algorithm. Next, based on annotated guidelines and manual observations on the training and development datasets, several pattern-based rules were designed and used in the postprocessing steps:

1. Rules were defined to distinguish between REACTION_COMPOUND and OTHER_COMPOUND based on context. Take the text in Figure 3 as an example. The first two chemical entities were labeled OTHER_COMPOUND because they were directly related to the entire process of “EXAMPLE 48” (i.e., REACTION_COMPOUND), while the main topic of the text snippet was to describe the first substep in the chemical reaction process—“48.1.” In contrast, the last chemical entity was labeled REACTION_COMPOUND because it was the target chemical in substep “48.1.”
2. The words “example,” “step,” “intermediate,” “core,” and “reference example”; brackets; square brackets; and curly brackets were removed from the EXAMPLE_LABEL type entity (e.g., Example 95).
3. The words “compound,” “example,” and “immediate” were removed from chemical entities in numerical patterns or patterns with mixed number and letter (e.g., compound 1-0003, Example 56A).

#### Subtask 2—Event Extraction

This subtask contains two steps. For the step of event trigger detection, it was also a NER task and was addressed with a similar approach as in subtask 1. For the relation extraction task, given the entities annotated in sentences, it can be transformed into a classification problem. A classifier can be built to determine categories of all possible candidate relation pairs (e1, e2), where entities e1 and e2 are from the same sentence. We generated candidate pairs by pairing each event trigger and semantic role. In order to represent a candidate event trigger and semantic role pair in an input sentence, we used the semantic type of an entity to replace the entity itself. The mentions of entities are directly generalized by their semantic types in the sentences. A linear classification layer was added on top of the Patent_BioBERT model to predict the label of a candidate pair in sentential context. As mentioned above, Patent_BioBERT was essentially built on the basis of BERT. In detail, BERT adds a classification token (CLS) at the beginning of a sentence input, whose output vector was used for classification. As typical with BERT, we used a (CLS) vector as input to the linear layer for classification. Then a softmax layer was added to output labels for the sentence.

In addition, some event triggers and their associated semantic roles appeared in different sentences or in different clauses in long compound sentences. Their relations were not identified using deep learning-based models. Therefore, postprocessing rules were designed based on the patterns observed in the training data and applied to recover some of these false-negative relations:

1. In the preprocessing stage, sentences/clauses containing “sat. aq.” (sat. aq. stands for saturate aqueous) were mistakenly segmented into two parts. For example, the clause “the residue was dissolved in EtOAc and washed with sat. aq. NH_4_Cl” was segmented into two parts—“the residue was dissolved in EtOAc and washed with sat. aq.” and “NH_4_Cl.” Such segments were merged to restore the correct clause, and then a relation was created between the nearest event trigger and the chemical behind “sat. aq.” Namely, a relation was created between “dissolved” and “NH_4_Cl” in the example sentence.
2. Some yield amount and percentage information were described in a separate sentence such as “Yield: 15 mg (25% of theory).” Relations were created to link them to the event trigger in the previous sentence, such as “purified” in “The mixture is purified by RP-HPLC (modifier: ammonium hydroxide).”

#### Subtask 2—End-to-End Extraction of Chemical Reaction

Overall, a typical cascade or pipeline model was built for the end-to-end system, in which semantic roles and event triggers were first recognized together in a NER model; their relations were then classified in a relation extraction model.

### Evaluation

Precision, recall, and F1 were used for performance evaluation, as defined in Eqs 1–3. Both exact and inexact (relax) matching results are reported. The primary evaluation metric was the F1 score of exact matching (He et al., 2020).

$$\mathrm{precision}=\frac{true\;positives}{true\;positives+false\;positives\;}$$ (1)

$$\mathrm{recall}=\frac{true\;positives}{true\;positives+false\;negatives}$$ (2)

$$F1=\frac{2\times precision\times recall}{precision+recall}$$ (3)

To determine which existing pre-trained language model should be fine-tuned and generate the patent language model, a pilot study was first conducted to compare the performances of different pre-trained language models, including BERT (Devlin et al., 2018), RoBERTa (Liu et al., 2019), Electra BERT (Clark et al., 2020), BioBERT (Lee et al., 2020), and Clinic BERT (Alsentzer et al., 2019). Models of NER and relation extraction were trained and tested on a small set of data, by fine-tuning different pre-trained language models. Based on our pilot experiments on 500 named entities, the NER F1s of 10-fold cross validation using BERT, RoBERTa, Electra BERT, and Clinic BERT were lower than that of BioBERT by 0.56, 0.59, 0.75, and 0.61, respectively. Experimental results demonstrated that fine-tuning BioBERT obtained the optimal performance. Therefore, in the rest of the work, we mainly used BioBERT as the basis to train the language models of chemical patents.

We used 10-fold cross-validation on the merged training and development datasets to optimize parameters for the models. The final set of hyperparameters and values used in the study are dropout_rate 0.2, max_seq_length 310, hidden_dim 128, learning rate 5e−5, and batch size 24. Based on this, we implemented three approaches for comparison:

1. Fine-tuning: Among the 10 models generated in the 10-fold cross-validation by fine-tuning Patent_BioBERT, the model with the highest performance on onefold was selected and used for submission.
2. Ensemble of output results from 10 models generated from the cross-validation using majority voting.
3. Merge-data: fine-tuning Patent_BioBERT using the merged training and development datasets.

Since the gold-standard annotations on the test dataset were not released, the performances of semantic role recognition and event recognition on the development data were reported for a detailed investigation.

In addition, we also checked the contribution from different strategies by implementing them incrementally into the model training process. Since the two-step tokenization mainly influenced the NER performance, the following were carried out:

1. BioBERT: BioBERT was fine-tuned for information WordPiece, following the original tokenizer in BERT.
2. Step tokenization for chemical patents: as mentioned before, we implemented an additional tokenizer to handle chemical patent text before feeding it into BioBERT, where it was further processed by WordPiece so that it can be aligned with the built-in vocabulary.
3. Postprocessing with pattern-based rules: once predictions are obtained from deep learning models, pattern-based rules are applied to postprocess and modify the predicted labels.

The above three steps construct a complete pipeline for information extraction. The following three strategies were further implemented to improve the performance:

4. Features of Word2Vec embeddings trained on a collection of 84K chemical patents shared from the ChEMU (He et al., 2020) were added, by concatenating them directly after embeddings generated from BioBERT.
5. Patent_BioBERT-ChEMU pre-trained on the training/development data: The BioBERT was replaced with Patent_BioBERT as the pre-trained language model, which is fine-tuned for models of information extraction.
6. Patent_BioBERT-External pre-trained on the training/development data and external patent data with chemical reactions: another version of Patent_BioBERT pre-trained with additional external text of chemical reactions was fine-tuned for models of information extraction.

## Results

Performances on the test dataset for each task are listed in Tables 1–3. Performances of the three approaches (Fine-tuning, Ensemble, and Merge-data) are reported for NER—recognition for semantic roles and event triggers in Table 1 and for event extraction in Table 2. The Fine-tuning approach outperforms the other two with the highest F1 of 0.957 for NER and the highest F1 of 0.9536 for event extraction. Therefore, only the Fine-tuning approach was used for building the end-to-end system, and its performance is reported in Table 3, with an F1 of 0.9174. The performances are top ranked based on the official evaluation in ChEMU (Nguyen et al., 2020).

**TABLE 1:** Performances of semantic role extraction for chemical reaction. Both exact and relaxed matching results are reported.

Method	Exact			Relax		
	Precision	Recall	F1	Precision	Recall	F1
Fine-tuning	0.9571	0.957	0.957	0.969	0.9687	0.9688
Ensemble	0.9587	0.9529	0.9558	0.9697	0.9637	0.9667
Merge-data	0.9572	0.951	0.9541	0.9688	0.9624	0.9656

**TABLE 2:** Performances of event extraction for chemical reaction. Both exact and relaxed matching results are reported.

Method	Exact			Relax		
	Precision	Recall	F1	Precision	Recall	F1
Fine-tuning	0.9568	0.9504	0.9536	0.958	0.9516	0.9548
Ensemble	0.9619	0.9402	0.9509	0.9632	0.9414	0.9522
Merge-data	0.9522	0.9437	0.9479	0.9534	0.9449	0.9491

**TABLE 3:** Performances of end-to-end systems for chemical reaction extraction. Both exact and relaxed matching results are reported.

Method	Exact			Relax		
	Precision	Recall	F1	Precision	Recall	F1
Fine-tuning	0.9201	0.9147	0.9174	0.9319	0.9261	0.9290

Interestingly, NER performances of the exact and relaxed matching criteria did not have sharp differences (Table 1), validating the effect of adding an additional tokenization step specifically for chemical patents in the preprocessing stage. However, the ensemble systems and systems built on the merged data of training and development sets yielded lower performances than the system fine-tuned on a 90%–10% split of the gold standard data. Especially, the Merge-data systems got the lowest performances among all three approaches. One potential reason was that the hyperparameters used in the Fine-tuning model were not the optimal set for the merged data.

Moreover, the detailed performances of the Fine-tuning method on the development set are reported. Table 4 lists the performances of each entity type (i.e., semantic roles and event triggers) and the overall performances. The overall F1 is 0.942 for NER. All the entity types yielded F1 scores above 90%. The lowest F1 scores were produced by the REACTION_PRODUCT (0.902) and the STARTING_MATERIAL (0.911). Both were frequently misclassified as OTHER_COMPOUND. Some STARTING_MATERIAL were also confused with REAGENT_CATALYST. As for the event triggers, REACTION_STEP obtained an F1 of 0.948, and WORKUP obtained an F1 of 0.931. They were often confused with each other when there was no clear transition from the reaction processing step to the later workup step. In the future, if semi-structures would be applied in patent writing with subsection headers like “Reaction:” and “Workup:,” these two processes could be differentiated straightforwardly. Interestingly, performances of the exact and relaxed matching criteria did not have sharp differences, which indicated that limited boundary errors occurred in the NER step. This validated that the preprocessing modules in CLAMP could efficiently segment sentences and split tokens.

**TABLE 4:** Performances on NER of semantic roles and event triggers on the development set are reported. The fine-tuning method was used in the experiment. Event triggers are italic.

Entity type	Exact		
	Precision	Recall	F1
EXAMPLE_LABEL	0.979	0.986	0.982
REACTION_PRODUCT	0.899	0.904	0.902
STARTING_MATERIAL	0.896	0.926	0.911
YIELD_OTHER	0.99	0.965	0.977
YIELD_PERCENT	0.972	1	0.986
REAGENT_CATALYST	0.938	0.905	0.921
SOLVENT	0.963	0.93	0.946
TEMPERATURE	0.935	0.96	0.947
OTHER_COMPOUND	0.947	0.939	0.943
TIME	0.983	0.991	0.987
REACTION_STEP	0.952	0.944	0.948
WORKUP	0.931	0.93	0.931
Overall_Semantic_Role	0.949	0.937	0.943
Overall	0.943	0.941	0.942

Table 5 lists the performances of each event type and the overall performance. The overall F1 is 0.953 for event extraction. Some rare events had lower performances, such as the ARG1 relation between REACTION_STEP and OTHER_COMPOUND (0.767) and between WORKUP and SOLVENT (0.333). Some events were described across sentences and were not covered by the current models, such as the ARGM relation between WORKUP and YIELD_OTHER and between WORKUP and YIELD_PERCENT.

**TABLE 5:** Performances on each relation type and the overall performance on the development set are reported. The fine-tuning method was used in the experiment.

Relation type	Exact		
	Precision	Recall	F1
ARG1|REACTION_STEP|OTHER_COMPOUND	0.733	0.805	0.767
ARG1|REACTION_STEP|REACTION_PRODUCT	0.985	0.948	0.966
ARG1|REACTION_STEP|REAGENT_CATALYST	0.979	0.965	0.972
ARG1|REACTION_STEP|SOLVENT	0.975	0.9522	0.968
ARG1|REACTION_STEP|STARTING_MATERIAL	0.957	0.916	0.936
ARG1|WORKUP|OTHER_COMPOUND	0.965	0.961	0.963
ARG1|WORKUP|REACTION_PRODUCT	0	0	0
ARG1|WORKUP|SOLVENT	0.2	1	0.333
ARG1|WORKUP|STARTING_MATERIAL	0	0	0
ARGM|REACTION_STEP|TEMPERATURE	0.957	0.928	0.942
ARGM|REACTION_STEP|TIME	0.978	0.926	0.952
ARGM|REACTION_STEP|YIELD_OTHER	0.984	0.942	0.962
ARGM|REACTION_STEP|YIELD_PERCENT	0.982	0.943	0.962
ARGM|WORKUP|TEMPERATURE	0.893	0.909	0.901
ARGM|WORKUP|TIME	0.7	1	0.824
ARGM|WORKUP|YIELD_OTHER	0	0	0
ARGM|WORKUP|YIELD_PERCENT	0	0	0
Overall	0.963	0.944	0.953

Table 6 lists the performances of semantic role recognition on the development and test data by implementing different methods incrementally. The performance change by adding each additional strategy is also reported. Starting from the baseline of fine-tuning on BioBERT (development: 0.8942, test: 0.9136), an additional step of tokenization yielded the highest performance improvement (development: 4.05%, test: 3.86%). Notably, applying language model features yielded different effects on the development and test data: (1) concatenating the Word2Vec embeddings pre-trained on chemical patents got an improvement of only 0.10% on the development data, while an improvement of 0.58% was obtained on the test data; (2) replacing BioBERT with Patent_BioBERT pre-trained on the training and development data (Patent_BioBERT-ChEMU) yielded an improvement of 0.47% on the development data. In contrast, the test performance dropped by 0.30%; (3) replacing BioBERT with Patent_BioBERT pre-trained on the training and development data and the externally collected patent data (Patent_BioBERT_External) dropped the performance on the development data by 0.32%. On the contrary, the test performance increased by 0.37%.

**TABLE 6:** Performances of semantic role recognition by adding different strategies incrementally. Exact matching results of the development and test data using the fine-tuning method are reported.

Model	Development			Test		
	Precision	Recall	F1	Precision	Recall	F1
BioBERT	0.8402	0.9556	0.8942	0.8587	0.9760	0.9136
+2Step_Tokenization	0.9364	0.9330	0.9347 (+4.05%)	0.9514	0.9530	0.9522 (+3.86%)
+Rule	0.9394	0.9351	0.9373 (+0.26%)	0.9539	0.9554	0.9546 (+0.24%)
+Word2Vec	0.9373	0.9394	0.9383 (+0.10%)	0.95984	0.9609	0.9604 (+0.58%)
+Patent_BioBERT-ChEMU	0.9491	0.9370	0.9430 (+0.47%)	0.9645	0.9517	0.9574 (−0.30%)
+Patent_BioBERT_External	0.9413	0.9383	0.9398 (−0.32%)	0.9616	0.9605	0.9611 (+0.37%)

Based on the experimental results, the Word2Vec embeddings and the language model of Patent_BioBERT_External, which were built on large collections of chemical patents had stronger generalizability on the test data. In contrast, Patent_BioBERT-ChEMU was overfitted on the development data which affected the generalizability on the test data. However, since we could only select the best models based on the development data during the participation of ChEMU, all submissions to the task were based on the language model of Patent_BioBERT-ChEMU, which actually yielded a lower performance on the test data.

## Discussion

Novel compound discovery is vital in the chemical and pharmaceutical industry. Chemical reaction is essential to the rigorous understanding of compound for further research and applications. Our participation in the CLEF 2020—ChEMU Task answers the urgent call for high-quality information extraction tools for chemical reaction information in patents. Evaluation based on the open test dataset demonstrated that the proposed hybrid approaches are promising, with top ranks in the two subtasks. Valuable lessons are also learned in this process:

A detailed error analysis was conducted for future system improvement. One major type of errors was the confusion between STARTING_MATERIAL and OTHER_COMPOUND, REAGENT_CATALYST and STARTING_MATERIAL, or REAGENT_CATALYST and SOLVENT. Information structures in sentences and context were not sufficient to differentiate these semantic types. Another major error was related to the event trigger recognition. Many false-positive event triggers were recognized, and REACTION_STEP and WORKUP were often confused with each other, especially for words frequently present in different contexts (e.g., added and stirring). Failing to recognize named entities correctly also affected the next relation extraction step. As for relation extraction, the majority of errors were caused by long-distance relations in intra- or inter-sentences.

The motivation behind the three implemented approaches is that it is interesting to examine if there is a space of performance improvement if majority voting or a larger training dataset is used. The three methods shared the same set of hyperparameters. However, the same set of hyperparameters, based on our current interpretation, is a curse to the final performances. The Ensemble and Merge-data approaches did not generate better performances as originally expected. More investigations need to be conducted for both, with an additional validation set for fine-tuning. Yet, the sensitivity of the hyperparameters in deep learning models is a long-standing problem that needs even more efforts to be alleviated.

Chemical tokenization has been an issue for long, and many efforts in the community were made to improve it, generating dedicated tokenizers such as Chemtok, Oscar4, and Umlsgenechem. In a previous work of chemical NER in patents, Zhai et al. (2019) made a comparison and demonstrated that chemical-specific tokenizers have a positive impact on NER performance. As mentioned previously in the method section, we also customized the tokenizer in CLAMP for chemicals and numerical values in patent text in this study. However, instead of focusing on exhaustively enumerating and capturing their structures in the tokenizer, we used punctuation as a separator to split the entire mention string into the smallest tokens, so that the tokenization results can be consistent for chemicals and numeric values. Our assumption was that the boundaries of NEs would be recognized automatically in a data-driven way, which was validated in the experimental results in Table 6. For illustration, the results of using CLAMP, Chemtok (Akkasi et al., 2016), Oscar4 (Jessop et al., 2011), and Umlsgenechem (NBIC, 2021) were shown in Table 7. As can be seen, the four tokenizers generated different results for the same chemical. Moreover, Umlsgenechem mistakenly removed the punctuation “%” in the percentage “58%” of yield material, and Chemtok identified the equation “J = 8.42 Hz” as a whole. In the next step, it is worth studying the performance of these tokenizers on patent datasets in different chemical subdomains (Zhai et al., 2019; Zhang et al., 2016; Krallinger et al., 2015b).

**TABLE 7:** Tokenization outputs of four tokenizers: CLAMP, chemtok, oscar4, and umlsgenechem.

Tokenizer	Chemical	Numeric values
Preparation of 5-formyl-2-trifluoromethylbenzonitrile	14.9 mg (58% yield); J = 8.42 Hz
CLAMP	Preparation of 5-formyl-2-trifluoromethylbenzonitrile	14.9 mg (58% yield); J = 8.42 Hz
chemtok	Preparation of 5-formyl-2-trifluoromethylbenzonitrile	14.9 mg (58% yield); J = 8.42 Hz
oscar4	Preparation of 5-formyl-2-trifluoromethylbenzonitrile	14.9 mg (58% yield); J = 8.42 Hz
umlsgenechem	Preparation of 5-formyl-2-trifluoromethylbenzonitrile	14.9 mg 58 yield; J = 8.42 Hz

Comparisons between the performances with and without postprocessing rules showed that the applied rules contribute only modest improvements to the overall performances. When the postprocessing rules were applied after the fine-tuning method, the exact-match F1s of NER, event extraction, and the end-to-end system were increased by 0.26% (0.9347 vs. 0.9373), 0.08% (0.9526 vs. 0.9534), and 0.24% (0.8946 vs. 0.8970) on the development set, respectively. In addition, the NER, event extraction, and end-to-end F1s were increased by 0.24% (0.9522 vs. 0.9546), 0.07% (0.9529 vs. 0.9536), and 0.22% (0.9152 vs. 0.9174) on the test set, respectively. Although rules were applied to fix errors from model predictions, they also brought false-positive instances. The precision and recall were examined carefully and balanced for each rule; only rules that could improve the performance with high confidences were kept in the system.

The experimental results demonstrated the fundamental importance of tokenization in the preprocessing step in adaptation to the unique characteristics in chemical patent text. Self-supervision based on larger-scale data achieved a stronger generalizability power of the pre-trained language models. Besides, more investigations are needed for heuristics and knowledge-based improvement.

### Limitations and Future Work

Although the proposed approaches obtained promising performances of chemical reaction extraction, there are several limitations. First, the number of documents used for experiments is limited, with a total of 180 patents for training, development, and testing. In addition, as the ChEMU organizers have noticed, there is a high degree of overlap between patents for training, development, and test sets. The expansion of the dataset, especially for open testing, will give a more comprehensive understanding of how the model performs in a more realistic setting and how to further improve the model. Next, domain knowledge of different semantic roles and their relations was not leveraged in the current study, such as lexicons of REAGENT_CATALYST and SOLVENT. This may potentially resolve the confusion among different semantic labels. Moreover, dependency syntactic information was not applied in the current approaches, such as conjunctive structures and header-dependent patterns. Such information was proven to be effective for relation extraction and would be integrated into the deep learning models to further improve the performance. In addition to tokenization, other basic building blocks of the pre-trained language model, such as vocabulary and representation of out-of-vocabulary tokens, will also be customized for chemical patents in the future.

## Conclusion

This work describes the participation of the Melax Tech team on the CLEF 2020—ChEMU Task of Chemical Reaction Extraction from Patent. We developed hybrid approaches combining tailored preprocessing, deep learning models, and pattern-based rules for this task. Our approaches achieved state-of-the-art results in both subtasks, indicating that the proposed approaches are promising. Further improvement will also be conducted in the near future by integrating domain knowledge and syntactic features into the current framework. Data augmentation will also be investigated for annotation enrichment in a cost-saving way, to further improve the system generalizability.

## Data Availability

Publicly available datasets were analyzed in this study. This data can be found here: http://chemu2020.eng.unimelb.edu.au/.
